# Patterns in clinical students’ self-regulated learning behavior: a Q-methodology study

**DOI:** 10.1007/s10459-016-9687-4

**Published:** 2016-05-27

**Authors:** Joris J. Berkhout, Pim W. Teunissen, Esther Helmich, Job van Exel, Cees P. M. van der Vleuten, Debbie A. D. C. Jaarsma

**Affiliations:** 1 Center for Evidence-Based Education, Academic Medical Center (AMC-UvA), University of Amsterdam, Amsterdam, The Netherlands; 2 Department of Educational Development and Research, Faculty of Health, Medicine and Life Sciences, Maastricht University, Maastricht, The Netherlands; 3 Department of Obstetrics and Gynecology, VU University Medical Center, VU University Amsterdam, Amsterdam, The Netherlands; 4 Institute of Health Policy and Management (BMG), Erasmus University, Rotterdam, The Netherlands; 5 Center for Research and Innovation in Medical Education, University Medical Center Groningen, University of Groningen, Groningen, The Netherlands

**Keywords:** Clinical learning, Q-methodology, Self-regulated learning, Undergraduate medical education

## Abstract

Students feel insufficiently supported in clinical environments to engage in active learning and achieve a high level of self-regulation. As a result clinical learning is highly demanding for students. Because of large differences between students, supervisors may not know how to support them in their learning process. We explored patterns in undergraduate students’ self-regulated learning behavior in the clinical environment, to improve tailored supervision, using Q-methodology. Q-methodology uses features of both qualitative and quantitative methods for the systematic investigation of subjective issues by having participants sort statements along a continuum to represent their opinion. We enrolled 74 students between December 2014 and April 2015 and had them characterize their learning behavior by sorting 52 statements about self-regulated learning behavior and explaining their response. The statements used for the sorting were extracted from a previous study. The data was analyzed using by-person factor analysis to identify clusters of individuals with similar sorts of the statements. The resulting factors and qualitative data were used to interpret and describe the patterns that emerged. Five resulting patterns were identified in students’ self-regulated learning behavior in the clinical environment, which we labelled: Engaged, Critically opportunistic, Uncertain, Restrained and Effortful. The five patterns varied mostly regarding goals, metacognition, communication, effort, and dependence on external regulation for learning. These discrete patterns in students’ self-regulated learning behavior in the clinical environment are part of a complex interaction between student and learning context. The results suggest that developing self-regulated learning behavior might best be supported regarding individual students’ needs.

## Introduction

Self-regulated learning (SRL) behavior is essential for future doctors’ life-long learning in their clinical environment (Ericsson 2015; Sandars 2009). Many undergraduate students struggle with SRL and feel insufficiently supported because the primary aim of health care is to provide care to patients, rather than to educate students (Teunissen and Westerman 2011). Furthermore, students may have difficulty fitting into their new roles as an aspiring doctor and learner in the clinical environment, and coping with the unpredictability of the clinical environment (Prince et al. 2005). As a result, students employ diverse, more or less self-regulated learning strategies that vary in effectivity, including proactively modulating affective, cognitive and behavioral processes to direct their learning in the clinical environment (Bjork et al. 2013; Paris et al. 2001; Sitzmann and Ely 2011; Zumbrunn et al. 2011).

Theoretically, SRL consists of a cyclical process initiated by goal setting, followed by deciding and implementing a strategy to achieve that goal, monitoring progress towards that goal and reflecting on the process afterwards and formulating new goals (Sandars and Cleary 2011). SRL is a process of the individual, but it is not an individualistic endeavor, being inextricably linked to learning context (Artino et al. 2011, 2012; Brydges and Butler 2012; Butler et al. 2011). A growing number of studies on SRL has focused on more complex contexts such as the clinical workplace (Artino et al. 2012; Berkhout et al. 2015; Brydges and Butler 2012; Durning et al. 2011; McEwen et al. 2015; Sagasser et al. 2012, 2015; Woods et al. 2011). Recent research noted that large individual differences existed in students’ SRL behaviors in clerkships (Berkhout et al. 2015). However, much about these individual differences is still unknown, such as whether distinct patterns exist and whether different individuals require different types of support. To best support students’ learning in the clinic it is important to gain a more thorough understanding of how students’ self-regulate their learning in the clinical environment, and to know if differences in learning behavior reflect distinct patterns in students’ SRL behavior. With this knowledge, better strategies to support students’ self-regulated learning in the clinical environment can be developed.

To improve our understanding of SRL behavior in the clinical environment, we formulated the following research question: what patterns in students’ self-regulated learning behaviors in the clinical environment can be identified, and what are their most important characteristics? We studied this by using Q-methodology (Watts and Stenner 2012), asking clinical students to sort a set of statements about SRL behaviors. This methodology has been advocated as a more robust technique than alternative methods such as Likert-type measurement scales to study attitudes in health education (Cross 2005).

## Methods

### Setting

We conducted this study in the Academic Medical Center in Amsterdam, the Netherlands, between December 2014 and April 2015. Students in the clinical phase of undergraduate medical education were eligible to participate (year four to six in an undergraduate-entry program). The preclinical curriculum is a block-based curriculum, with vertical integration of preclinical and clinical sciences and horizontal integration of basic knowledge and skills into the clinical context. Clerkships consist of 13 discipline-based rotations in clinical departments, outpatient clinics and community settings, lasting for 2–10 weeks.

### Participants

Considering the importance of the interaction between individual and context for self-regulated learning behavior, we aimed for maximum variability by including students who were enrolled in various stages of the clerkships in either the academic hospital, its affiliated regional hospitals or in a community setting (Butler et al. 2011). We only included students who had participated in more than three different clerkships, because we assumed it takes time and experience for students to gain insight in their own SRL behavior in the clinic. We set no specific exclusion criteria. Eligible students were approached in groups to participate. These groups were based on date of initial enrolment in the clerkships, representing varying stages of advancement through the clerkships to ensure diversity in experience, and on current enrollment in different clerkship specialties to ensure diversity in the current learning context. There is no definite number of participants required in a Q-methodology study. In general, 40–60 participants is considered to be adequate (Watts and Stenner 2012, p. 73). However, because we sampled groups of students rather than individuals and we wished to minimize the risk of missing a less prevalent behavior pattern, we aimed for a higher number of approximately 75 participants out of a total of approximately 800 eligible participants. Groups of students were approached one after another either by email or in person to invite them to participate in the study until we had reached our aim of approximately 75 participants. As an incentive, a lottery decided which two students won a gift certificate.

### Q-methodology

The Q-methodology has been used before in the context of medical education (Fokkema et al. 2014; Gaebler-Uhing 2003; Meade et al. 2013; Wallenburg et al. 2010). It uses features of both qualitative and quantitative methods for the systematic investigation of subjective issues (Barbosa et al. 1998; Cross 2005; Watts and Stenner 2012). It is important to include participants who represent a variety of perspectives in the sample. Participants are instructed to sort a set of statements along a chosen continuum on a fixed grid. Next, they are asked to explain their sorting of the statements. This narrative information supports the interpretation of the quantitative findings and is used for elucidation of the results. Patterns are identified in the sorting of statements using by-person factor analysis (Kline 1994). The resulting patterns are interpreted and described as shared perspectives on the subject of study.

### Study design

**Fig. 1:**
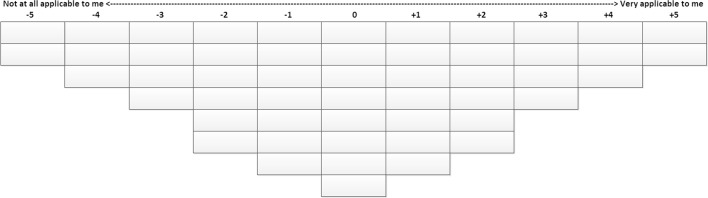
Representation of the grid used in the online sorting procedure. Participants assigned all 52 statements to one of the places on the grid, representing how well each statement described their learning behavior

We provided students with statements on SRL behaviors in the clinical environment (see Table 1) and asked them to evaluate these statements according to how well they describe their learning behavior. The students sorted the statements along a continuum ranging from “not at all applicable to me” to “very applicable to me” on a fixed grid as shown in Fig. 1. The study was administered through a web application (www.qsortouch.com).

Within the web application, we asked students for their informed consent and their basic demographic details. Next, we presented the statements one-by-one in random order and asked students to sort them into one of three groups: not applicable to me, neutral, and applicable to me, without limiting the number of statements that could be assigned to any group (Watts and Stenner 2012). We subsequently asked the students to assign the statements in each group to one of the places on the sorting grid. As a final step, we asked the students to elaborate on the reasons why the statements placed at the extreme ends of the grid were most and least applicable to their learning behavior, and what they felt is characteristic for their learning in the clinical environment.

### Statement set

**Fig. 2:**
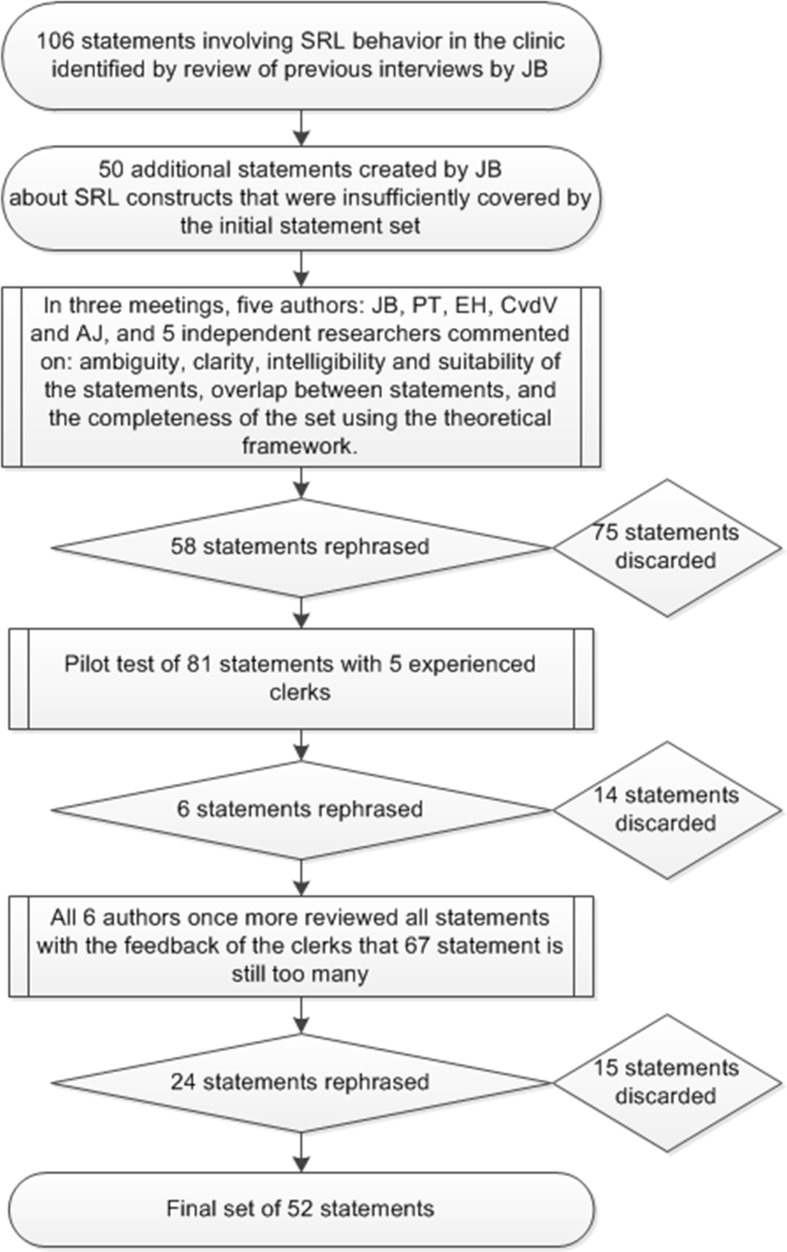
Flowchart depicting the process leading to the final set of statements used

The primary researcher (JB) revisited the transcripts of an earlier interview study on students’ SRL in clinical environments to create the initial set of statements (Berkhout et al. 2015). In this prior study we interviewed 17 students’ from two different Dutch universities about their SRL behavior. This data was especially suitable for the current study because it gave us access to behavioral descriptions provided by students themselves. To make sure the statement set covered all relevant aspects of SRL, we used a framework of 16 fundamental constructs that defined self-regulated learning in the seven most cited SRL theories (see Box 1) (Sitzmann and Ely 2011). This led to an initial set of 156 statements. We discussed the statement set within the research team during three meetings. In the first meeting statements were rephrased or discarded whilst focusing on the ambiguity, clarity, and suitability of the statements. In the second meeting, statements were discarded or rephrased with a focus on intelligibility, overlap between statements, and completeness of the set which was informed by the feedback of 5 unassociated researchers in the field of medical education. In the third meeting, the research team discussed the statements whilst making sure all 16 constructs were covered by at least three statements and lowering the number of statements for the sorting procedure to a more manageable 82. This set was then piloted in 5 experienced clerks. Based on their feedback in a final meeting with the research team we agreed upon a final set of 52 statements. Figure 2 provides a visual representation of the development of the statement set. The statements were originally in Dutch, and were translated by the authors for this paper.

**Box 1:** Sitzmann and Ely’s description of the 16 constructs that are included in the seven most cited theories on SRL (Sitzmann and Ely 2011)

Goal level	Standards trainees aim to achieve during training
Planning	Thinking through what one needs to learn, setting task-specific goals, and deciding which strategies to employ to achieve the goals
Monitoring	Paying attention to one’s performance and understanding of the course material
Metacognition	Planning and monitoring goal-directed behavior and devoting attention toward the course material
Attention	Concentrating and maintaining one’s mental focus during training
Learning strategies	Techniques employed to elaborate on the training material as well as integrate all of the components of the material with each other and with one’s existing knowledge
Persistence	Continuing to allocate effort and attention toward the training material, despite boredom or failure to make progress toward one’s goals
Time management	Making study schedules and allocating time for study activities
Environmental structuring	Choosing a study location that is conducive to learning
Help seeking	Seeking assistance when one has difficulty understanding concepts during training
Motivation	Willingness to engage in learning and desire to learn the course content
Emotion control	Keeping negative emotions at bay while learning
Effort	The amount of time that trainees devote to learning
Self-evaluation	Assessing goal progress by comparing one’s current level of knowledge or performance with the desired goal state
Attributions	Trainees’ beliefs about the causes of outcomes in achievement situations
Self-efficacy	Trainees’ beliefs regarding their capability to succeed in training and perform training-related tasks

### Analysis

We analyzed the data using dedicated software (PQMethod 2.11) to perform a by-person factor analysis to extract the statistical factors, corresponding to patterns in behavior (Schmolck and Atkinson 2002). This technique clusters individuals with similar answers together, rather than items, as is the case in traditional factor analysis. We conducted the analysis using common techniques for Q-methodology (centroid factor rotation and varimax rotation) (Watts and Stenner 2012). We identified all factor structures supported by the data by using the commonly used criteria of Eigenvalue >1.00, and a minimum of two participants who were associated statistically significantly (*p* < 0.05) with the factor (Watts and Stenner 2012). Three researchers (JB, PT, EH) studied and discussed the factor structures supported by the data to see whether the factors identified in each solution represented coherent patterns in SRL behavior. The final factor solution was selected because it consisted of five clearly interpretable and distinct factors that together portrayed a comprehensive summary of the (quantitative and qualitative) data.

Next, an idealized sort was computed for each of the retained factors. This is a weighted ranking of the statements for the students associated with the factor, based on their rankings of the statements and their correlation coefficients with the factor as weights. This idealized sort represents how a hypothetical student with exactly that pattern in SRL behavior would have ranked the statements, and serves as the basis for interpretation and description of the results. From the idealized sorts, distinguishing statements between factors and consensus statements across factors were identified. Distinguishing statements significantly differ in position in the idealized sort for a certain factor compared to all other factors, and consensus statements do not have a significantly different position for any factor. JB, PT and EH then interpreted all factors by using the idealized sorts, the distinguishing and consensus statements, and the narrative data from the participants significantly associated with each factor. Lastly, we provided a descriptive label to all factors for the ease of remembrance and future use.

### Ethics

The ethical review board of the Netherlands Association for Medical Education (NVMO) approved the study, file number 376. All participants provided informed consent.

## Results

We invited 203 medical students, of whom 74 (36 %) agreed to participate and completed the procedure. 29 participants were male, 45 were female. The mean age was 24.5, with a range of 22–39 years old. 22 participants were in year 4, 48 in year 5, and 4 in year 6 of the six-year undergraduate program. The participants were enrolled in 11 different clerkships: family medicine, internal medicine ward, internal medicine outpatient clinic, neurology, obstetrics/gynecology, pediatrics, psychiatry, social medicine, surgery outpatient clinic, surgery ward and various elective clerkships.

The 74 sorts of the statements supported a maximum of five patterns, which were retained as a final solution after analysis of compensability, clarity and distinctiveness of the resulting patterns. The five patterns explain 43 % of the total variance in the data. Each pattern was defined by 7–18 participants.

Table 1 presents the idealized sorts for each of the five patterns, as well as the distinguishing statements for each pattern. There were no consensus statements.

**Table 1:** Complete list of 52 Q-sort statements and idealized sorts for the five patterns representing students’ self-regulated learning behavior

Statement pattern	1: Engaged	2: Critically opportunistic	3: Uncertain	4: Restrained	5: Effortful
1 I do what my supervisor asks from me	+1	+2	+4	+5	+5
2 I deliberately plan my learning	+2	+1	−*3* ^*b*^	−*1* ^*b*^	*0* ^*b*^
3. If there is little to do on the ward, I will go and try to find other learning opportunities	+*4* ^*a*^	−*2* ^*b*^	−1	−1	+1
4. I ask myself questions when I am reading new information to test if I *really* know it	−*2* ^*b*^	−3	−4	+*1* ^*b*^	+*2* ^*b*^
5. I make sure I keep paying attention during educational talks	+2	+1	+3	+2	−*2* ^*a*^
6. I actively follow up on patients	+2	+*3* ^*b*^	+2	+3	+1
7. I am well prepared for a clerkship day	+1	−1	+2	−2	+*4* ^*a*^
8. I sometimes do something I do not think is useful, because it will enable me to do something different that I do think is useful	−3	−1	−1	−3	+*3* ^*a*^
9. I schedule the tasks I have to do in a day	+1	+1	0	+2	−*2* ^*b*^
10. I go to seek a computer elsewhere, if there is not one available for me in the doctors’ room	+1	+2	+3	+4	−*1* ^*a*^
11. I only ask for help if I do not see *any* alternative	*0* ^*a*^	−1	−2	+*5* ^*a*^	+*3* ^*a*^
12. I work as independently as possible	+*1* ^*b*^	+*2* ^*b*^	−*3* ^*a*^	+*4* ^*b*^	+*5* ^*b*^
13. I find it difficult to motivate myself for specific clerkships	−*5* ^*a*^	+*3* ^*b*^	+2	0	+2
14. I try to appear enthusiastically	+5	+5	+*5* ^*a*^	+*1* ^*a*^	+4
15. I evaluate for myself how far I am in achieving my goals for a clerkship	−2	*0* ^*a*^	−*5* ^*b*^	−*5* ^*a*^	−3
16. What I do with feedback depends on who gave it to me	−*1* ^*b*^	+3	+1	+2	+1
17. I will tell my supervisor if I am insufficiently prepared	−3	*0* ^*a*^	−5	−4	−5
18. I ask my supervisors what they expect of me	0	+*4* ^*a*^	+1	−*5* ^*a*^	+1
19. I make sure I am useful to the department	+5	+4	+4	+3	+3
20. I prioritize which skills I want to gain	0	+1	0	−1	−1
21. I use the handovers to discover what I want to learn more about	−1	−*5* ^*a*^	+*1* ^*a*^	−2	−1
22.I think about how I can learn best in a specific department	0	−1	−2	0	−2
23. I actively engage in thinking about the cases presented during handovers	+1	−1	0	+1	+1
24. I use other students’ experiences to learn from	−*1* ^*a*^	+2	+*5* ^*a*^	+3	+2
25. Even if I have to do something I do not think is interesting, I still try to learn something from it	+*2* ^*b*^	−2	−1	0	0
26. I often continue work during the breaks	−4	−3	−2	−2	+*3* ^*a*^
27. I make sure I work in a place where I can stay focused	−1	0	+1	+1	−*3* ^*a*^
28. I ask my supervisor for advice on my learning goals for the clerkship	−2	−2	+1	−3	−1
29. I try to have a good time in my clerkship	+4	+4	+4	+*1* ^*b*^	0^*c*^
30. I reassure myself when I am nervous	−3	−4	+2	0	+1
31. I do more than other students during the clerkships	*0* ^*b*^	−3	−*1* ^*a*^	−4	+*2* ^*b*^
32. I ask others how they think about my performance	0	+1	+2	−*4* ^*a*^	0
33. If I learned little from something, I ask myself why that happened	−2	−4	−4	*0* ^*a*^	−2
34. If I think that something a supervisor says is wrong, I tell them what I think is correct	−2	+*2* ^*a*^	−*4* ^*a*^	−1	−3
35. I set medical knowledge learning goals for myself	0	0	−*2* ^*b*^	0	0
36. I deliberately choose which patients of the outpatient clinic I see	−3	−*5* ^*a*^	−2	−1	−1
37. I actively shape my learning	+*3* ^*a*^	−1	−1	−1	0
38. I can concentrate in a hectic clinical setting	+3	*0* ^*b*^	−2	+1	−3
39. I ask my supervisor questions	+3	+5	+3	+3	+4
40. I postpone tasks I do not like doing	−*4* ^*a*^	−2	0	−1	−2
41. When my day at the clerkship is over, I will not do anything more that has to do with medicine	−*4* ^*a*^	+1	+1	−*2* ^*a*^	−*5* ^*a*^
42. I set learning goals for myself regarding the communication with patients	−1	−1	−3	−3	−1
43. I actively seek feedback about my functioning to formulate learning goals for myself	+1	+1	−3	−3	−*1* ^*b*^
44. I work extra hard to improve skills I think are difficult	+3	−*2* ^*b*^	0	0	+2
45. I make sure I work with supervisors of whom I think I can learn a lot	+2	−3	+2	+1	−2
46. I ask other students about the normal habits in a specific department	−*1* ^*a*^	+3	+3	+2	+*1* ^*b*^
47. I get the most out of a clerkship	+*4* ^*a*^	*0* ^*b*^	−1	−2	+*2* ^*a*^
48. An emotional event influences me the rest of the day	−2	−4	*0* ^*a*^	−2	−4
49. I make sure there is a good balance between work and private life	+2	+2	*0* ^*a*^	+2	−*4* ^*a*^
50. I try to read from the reactions of others if I did perform well	−1	0	+1	+*4* ^*a*^	0
51. After having seen a patient, I think about how I could do (even) better next time	0	0	−1	+*2* ^*a*^	0
52. If I’m afraid I will do something wrong, I refrain from doing it	−*5* ^*a*^	−*2* ^*b*^	0	0	−*4* ^*b*^

The numbers ranging from −5 to +5 correspond to the location of the statements in an idealized sort representing each pattern, placed on a grid as is shown in Fig. 1. Distinguishing statements for each pattern are italicized

^a^Distinguishing statements *p* < 0.01

^b^Distinguishing statements *p* < 0.05

In the next section we give the statistical characteristics and our interpretation of the five patterns portraying self-assessed SRL behavior of clinical students. Between parenthesis, the illustrative statements for particular behavior are given, with the numbers ranging from −5 to +5 corresponding to the placement of the statements in the idealized sort of the factor, corresponding to the columns of the grid as is shown in Fig. 1. Each description of a pattern ends with illustrative quotes from the narrative data, where “R” followed by a number refers to the unique identifier of the respondent. Table 2 summarizes the findings.

**Table 2:** Summary of SRL behavior patterns

SRL behavior pattern	Characterized by:
1. Engaged	The student is highly self-regulating and learning oriented. The student is enthusiastic, hardworking, motivated, not afraid to make mistakes, and not easily affected by context
2. Critically opportunistic	The student interacts a lot, is enthusiastic, has little regard for hierarchy and wants to enjoy the clerkships. The student uses little effort, does not structure the learning environment, is critical of the learning environment, and can easily lose motivation
3. Uncertain	The student is overwhelmed by the clinical environment, needs a safe environment to learn and shows little self-regulation. The student behaves passively and is highly dependent on the supervisor
4. Restrained	The student is highly motivated, self-critical, but is afraid to appear inferior to others and therefore wants to learn independently. The student realizes the need for guidance from supervisors, but is afraid to ask questions and ask for feedback
5. Effortful	The student works very hard compared to peers and always comes prepared. The student needs to be told what to do, wants to learn independently, but shows little environment structuring and is afraid to admit being in difficulty

### Pattern 1

Pattern 1, which we labelled “Engaged”, was defined by the sorts of 6 male and 12 female students, explaining 13 % of the total variance. Students reporting to show this behavior pattern know where they want to go in their learning, and how to get there. They see themselves as enthusiastic (statement 14, +5), motivated (statement 13, −5), hardworking (statement 40, −4; statement 41, −4; statement 44, +3), and try to make themselves useful (statement 19, +5). They want to learn the most they can in their clerkships (statement 25, +2; statement 47, +4) and try to enjoy them (statement 29, +4). They describe to actively shape their learning (statement 2, +2; statement 3, +4; statement 37, +3) and seek little external regulation (statement 1, +1) or help from peers (statement 24, −1; statement 46, −1). They say they are able to function in difficult, hectic environments (statement 38, +3) and are not afraid to try something they are not sure they are capable of doing (statement 52, −5). This results in a behavior pattern in which students actively shape their learning, are motivated to learn from every situation and learn in a self-regulated fashion.

R3, a 23-year-old female enrolled in a pediatrics clerkship in the 4th year: “I work independently, I am assertive, and I take care of my own education (other people won’t do it for you)”

R56, a 39-year-old female enrolled in a psychiatry clerkship in the 5th year: “I am a self-critical student who actively tries to shape my clerkships. If I don’t understand something and think it is interesting/important, I will make a presentation about it to make sure I understand it thoroughly”.

### Pattern 2

Pattern 2, which we labelled “Critically opportunistic”, was defined by the sorts of 3 male and 5 female students, explaining 8 % of the total variance. In this behavior pattern, students feel that having a good time is important (statement 29, +4) and they report to present themselves enthusiastically to achieve this (statement 14, +5). They feel that learning often happens through social interaction with supervisors (statement 18, +4; statement 39, +5), and to a lesser extent with peers (statement 24, +2; statement 46, +3). Students disclose they do not put a lot of effort into their learning in the clerkships (statement 31, −3), and do not structure their learning environment (statement 3, -2; statement 36, −5; statement 45, −3). They are critical about the learning opportunities in a department (statement 16, +3; statement 21, −5) and report having no problems critically conversing with others higher in the department hierarchy (statement 17, 0; statement 18, +4; statement 34, +2). They tend to lose motivation if they are not having a good time or do not see the goal of a task (statement 13, +3; statement 25, −2). This results in a behavior pattern in which students are opportunistic in their learning, critical towards their environment, try to have a good time during their clerkships and seek interaction with supervisors.

R19, a 24-year-old female enrolled in an internal medicine clerkship in the 4th year: “I don’t deliberately choose the patients I see in the outpatient clinic (especially in the academic hospital, I’m glad if I get to see a patient on my own).I don’t work during brakes and make sure I take a brake every day”.

R65, a 24-year-old female enrolled in a general practice clerkship in the 5th year: “I won’t put in extra effort for something I don’t like”.

### Pattern 3

Pattern 3, which we labelled “Uncertain”, was defined by the sorts of 3 male and 5 female students, explaining 8 % of the total variance. Students assessing themselves according to this pattern seem to be overwhelmed (statement 48, 0) or even frightened by the clinical environment (statement 30, +2; statement 52, 0) and their supervisors (statement 34, −4). They try to make a good appearance towards supervisors (statement 14, +5; statement 17, −5) and hope they will have a good time (statement 29, +4). They describe to not actively shape their learning in the form of goal setting (statement 35, −2; statement 42, −3), planning (statement 2, −3), monitoring (statement 4, −3) or evaluating/reflecting (statement 15, −5; statement 33, −4; statement 43, −3). They reveal to heavily depend on their supervisor for their own learning (statement 1, +4; statement 45, +2) and do not learn very independently (statement 12, −3) nor actively search for learning opportunities (statement 3, −2). They explain their strategies for learning in the clinical environment mainly involve maintaining attention during safe, structured formal educational sessions (statement 5, +3), and peer learning (statement 24, +5; statement 46, +3). This results in a passive behavior pattern that seems to fit insecure students learning in a difficult, hectic clinical environment.

R5, a 24-year-old female enrolled in a community health clerkship in the 5th year: “If a department is very hectic/chaotic, I often have difficulty finding my place, finding out where I can help. I learn most from real cases. Unfortunately, not all supervisors have the time, or are willing to take the time to extensively discuss these cases with the students”.

R64, a 26-year-old female enrolled in a general practice clerkship in the 5th year: “I’m not good in actively giving my opinion or prioritizing. I learn by doing my best, without having predefined learning goals for myself”.

### Pattern 4

Pattern 4, which we labelled “Restrained”, was defined by the sorts of 4 male and 3 female students, explaining 8 % of the total variance. Students reporting this behavior pattern are characterized by rarely asking questions to supervisors or peers (statement 11, +5; statement 18, −5; statement 28, −3; statement 43, −3), especially if it can make them appear inferior to others (statement 17, −4; statement 32, −4; statement 52, 0). Rather, students explaining to use this behavior pattern try to learn as independently as possible (statement 12; +4), observe others, and interpret implicit reactions of others to judge their performance (statement 50, +4). They disclose to rely on their supervisors’ instructions (statement 1, +5), engage in little planned learning (statement 2, −1; statement 25, −5; statement 37, −1), and some reflective learning (statement 51, +2). These students are motivated for learning, less concerned with having a good time (statement 14, +1; statement 29, +1), and realize they could get more out of the clerkships than they are currently doing (statement 31, −4; statement 47, −2). This results in a behavior pattern in which students want to learn, but hesitate to include others in this process, following the instructions given by supervisors without asking question, as they fear to appear inferior.

R28, a 24-year-old female enrolled in a neurology clerkship in the 5th year: “I try to go with the flow, I often forget there are more opportunities to learn during a clerkship by broadening my view. Admitting I did something wrong or that I’m not prepared, is not something I would easily do, I’d rather hope it would go by unnoticed”.

R49, a 26-year-old male enrolled in an internal medicine clerkship in the 4th year: “I try to participate and be a good colleague, but I should be focusing more on my learning goals”

### Pattern 5

Pattern 5, which we labelled “Effortful”, was defined by the sorts of 3 male and 4 female students, explaining 6 % of the total variance. Students related to this self-assessed behavior pattern have the urge to work very independently (statement 12, +5) and to work hard (statement 7, +4; statement 31, +2; statement 49, −4), but rely on their supervisor to guide them (statement 1, +5) because they are unsure what they need to learn in their clerkships. These students explain they are likely to do everything they are asked (statement 8, +3) and mostly rely on effort to learn (statement 41, −5) whilst having difficulty structuring their environment to make it suitable for their learning (statement 9, −2; statement 27, −3; statement 38, −3). They want to appear very capable, and won’t easily admit being in difficulty (statement 11, +3; statement 17, −5). This results in a behavior pattern in which students work particularly hard and always come prepared, trying to function independently and leave a good impression, but rely heavily on the supervisor for guidance because they are not capable to structure their learning environment.

R13, a 25-year-old male enrolled in a surgery clerkship in the 4th year: “I learn on-the-go, if I come across something I don’t understand, I try to look it up as soon as I can, or ask someone. To me, working independently is especially important. My functioning as a doctor later will largely depend on a certain degree of independence. By showing enthusiasm I think I will learn most”.

R41, a 25-year-old male enrolled in an elective cardiothoracic surgery clerkship in the 6th year: “My learning is exemplified by me writing down all questions I have during the day, and then study in the evening”.

## Discussion

We identified five distinct patterns in students’ self-assessed self-regulated learning behavior to learn in the clinical environment. The patterns varied widely regarding goals, metacognition, communication, effort, and dependence on external guidance for learning and resulted from a complex interaction between individuals and the context they learn in. The clinical context is one in which it may be difficult to learn because students have a hard time knowing what they can expect and a hard time dealing with the unpredictability of the clinical environment (Prince et al. 2005). This is reflected to a varying extent in all SRL patterns by the poorly planned SRL behavior in general and limited goal setting specifically.

Sitzmann and Ely concluded that SRL constructs regarding goals and self-efficacy have the largest impact on the effect of SRL in a workplace and that metacognitive strategies (a combination of planning, monitoring, metacognition and learning strategies), attributions, effort, time management, motivation and environment structuring have a weak to moderate effect on SRL (Sitzmann and Ely 2011). In our study, the goal setting and high self-efficacy were most prominently notable in the engaged pattern and largely absent the uncertain pattern. Both the engaged and the critically opportunistic patterns prominently involved using metacognitive strategies. Using attributions for learning is prominent in the restrained pattern. However by wanting to perform, rather than learn, the effect of SRL may be impaired. Time management and effort on learning most prominently showed in the effort pattern. If the results from Sitzmann and Ely’s meta-analysis are also valid for the clinical context, this might mean the engaged pattern would lead to the best learning outcomes and the use of this behavior pattern should be encouraged.

Woods et al. have also looked at the self-regulated learning in a clinical context, specifically the informal aspects of SRL in a surgical clerkship, and discovered three separate “approaches” to SRL (Woods et al. 2011). The first approach: acquiescing to a lack of learning opportunities, relates to critically opportunistic pattern that we found. This approach also features a focus on contextual barriers on learning and subsequent frustration and loss in motivation. The second approach they found: choosing learning opportunities, covers important aspects of the restrained and effortful patterns we found. This approach also emphasizes how students believe a lot of effort is required to learn in the clinical environment and how they try to balance the demands their learning forms for the context and maintaining a good relationship with residents and staff. The third approach: creating learning opportunities, closely resembles the engaged pattern, where students are characterized by trying to make sure learning is maximized at every moment and is deemed favorable. In the study of Woods et al. self-reflection and a pattern of behavior dominated by uncertainty (pattern 3 in our study) were not prominently addressed by the students. Our study adds to this knowledge by including patterns in behavior regarding reflection and feedback, and gives an insight in additional ways how students self-regulate their learning in the clinical environment.

The patterns we have found in SRL behavior in the clinical context also show similarities with the “stances” in first-year undergraduate medical education described by Evensen et al. (2001). The six separate stances they found served to govern perceptions of the students themselves and their context. The interactive stance, which is described as being motivationally, metacognitive and behaviorally in charge of one’s own learning, shows many similarities with the engaged pattern. The proactive stance, in which a student is highly motivated and inventive, but inattentive to the certain affordances of an environment that could relieve burdens, shows many similarities with the critically opportunistic pattern. The retroactive stance is closely related to the proactive stance, but involves the use of ineffective learning strategies from a different context. This shows many similarities with the effort pattern where students solely rely on very high effort to support their learning. The reactive stance, where the students entirely relies on the context to guide their learning, has some similarities with the uncertain pattern, however the importance of low self-efficacy and not wanting to be a burden are emphasized even more in the pattern we found. The transactive stance, which is similar to the interactive stance but includes the student wanting to be a full and legitimate member of the team, did not clearly emerge from our data. This might be because in the clinical context students are frequently relocated, not allowing them to really become legitimate members of a clinical team. Contrarily, the stances described did not include a stance similar to the restrained pattern. This is again likely due to context. The stances theory was developed studying undergraduate students in a PBL curriculum. These students do not yet face the challenges of hierarchy and busy schedules of a clinical context and therefore do not show a clearly restrained learning pattern. These differences again highlight the importance of studying SRL in context (Butler et al. 2011).

Evensen et al. also noticed how their stances could be related to identity development (Evensen et al. 2001). Whilst interpreting the patterns resulting from our study, we also noted a resemblance between the patterns and theories on identity development. A resemblance between identity development in the clinical environment and our resulting patterns is understandable, because what students want to learn and what they find important might be the result of a students’ process of developing an identity in a clinical environment. This is coherent with theoretical beliefs that self-regulated learning, and regulation of behavior specifically, is a consequence of trying to convey a specific identity towards others and that students may experiment with possible alternative identities (Paris et al. 2001). Our findings might therefore provide an additional perspective to recent postulations that the development of a professional identity should be a principal goal of medical education (Cruess et al. 2014).

The main strength of our study is that the set of statements used was distilled from actual interviews with medical students, facilitating recognition by participants. The set was also structured and selected using an overarching theory on SRL, decreasing the chance of missing important aspects of SRL behavior. In light of the importance of the interaction between individual and context, another main strength of our study is having data from students from 11 different clerkships and in various stages of the clerkships, recruited from various hospitals and community settings. This greatly increased the chance of finding all relevant SRL behavior patterns present. The total variance explained by the five factors is 43 %, which is considered to be a sound factor solution in a Q-methodology study (Watts and Stenner 2012, p. 105).

Based on our study we can say that at least five different patterns in SRL behavior are described by students who are learning in the clinical environment. However, we are aware that curriculum pedagogy can influence students’ SRL (Lucieer et al. 2015; White 2007). It is therefore possible that other patterns, or a shift in patterns’ features, can be found when studying students from other medical schools. The online data collection procedure has limited the extent to which we gained a deep insight into the patterns. Using an in-person procedure might have helped understand what entices students to engage in a certain behavior pattern and to better understand what characteristics of individual and environment have an effect on SRL behavior. The individual influence that various contextual factors, such as societal and cultural factors, and personal factors, such as individual experiences, have on SRL behavior patterns remain unknown.

Nonetheless, what is evident form our results is that students have different patterns in their SRL behavior. Students describing these different behavior patterns are likely to require different forms of support to assist them in their self-regulated learning. Program directors and clinical supervisors may try to foster this by applying a more individual approach to supervision. For example, students who with an engaged SRL behavior pattern may be supported by allowing them relative high autonomy in clerkships (Berkhout et al. 2015). Students with a critically opportunistic SRL behavior pattern may be helped by making them understand why certain things need to be learned (Berkhout et al. 2015). Students with an uncertain, restrained or effortful SRL behavior pattern may benefit from discussing the goals they work towards. Especially, inspiring them to focus on learning rather than striving to appear competent or avoiding to appear incompetent (Vrugt and Oort 2008). Additionally, students with an uncertain or effortful SRL behavior pattern benefit from a safe and supportive learning environment (Van der Hem-Stokroos et al. 2003), and may be supported by engaging them in a discussion about how to learn in a clinical environment (Cornford 2002; Sandars 2010). Mentoring (Driessen and Overeem 2013), mapping (Patel et al. 2015), and a microanalysis of students’ current SRL process (Cleary and Sandars 2011; Cleary et al. 2012; Patel et al. 2015), are increasingly studied and seem promising strategies to help identify individual students’ SRL behavior patterns, enable feedback to be given on key SRL process, and subsequently achieve more personalized contextual support.

Our results suggest that SRL behavior and context are closely intertwined. Evensen et al. found the stances to initially evolve in a PBL context and usually result in a stable stance, however some students also varied in their stance throughout their course (Evensen et al. 2001). It would be interesting to conduct longitudinal research using in-depth interviews with people representing each identified pattern to enhance our current understanding of SRL behavior patterns in the clinic and how these vary or develop within students. Additional survey research using representative samples of students needs to be conducted to know how frequent the patterns are in the wider student population. Measuring SRL in the clinical environment, could quantify what effect the five SRL behavior patterns have on medical knowledge, and other competencies. This would further differentiate between more desirable and less desirable behavior patterns. Furthermore, it would be insightful to study if supervisors recognize the SRL behavior patterns and if they can use this recognition to personalize the support and guidance they provide to individual students. It would also be interesting to gain a deeper understanding of how various aspects of context influences SRL behavior and how these could be used to support more favorable SRL behavior. Lastly, it would be interesting to more closely study how identity development and self-regulated learning behavior are related, and how these can be supported to create high self-regulating, life-long learning physicians.

## Conclusions

We distinguished five patterns in clinical students’ reported SRL behavior; engaged, critically opportunistic, uncertain, restrained, and effortful. These patterns are part of the complex interaction between individual and context, and varied strongly regarding goals, self-efficacy, metacognitive strategies, effort, and relying on self- versus externally regulated learning behavior. These different patterns are likely to require different types of support to optimize the effect of SRL in the clinical. Therefore an individualized approach to supervising students learning in the clinical context needs to be taken. Mentoring, mapping and microanalysis protocols seem promising strategies for recognizing individual needs and individualizing the contextual support given.
